# Evaluation of an oral care bundle for reduction of nonventilator hospital-acquired pneumonia in a community hospital

**DOI:** 10.1017/ash.2022.172

**Published:** 2022-05-16

**Authors:** Elias Coury, Shannon Dietz

## Abstract

**Objectives:** We sought to determine the relationship between an oral-care bundle that includes use of new oral care devices, education of best practices for performing oral care, and daily audits to measure compliance with oral care best practices and the reduction of nonventilator hospital-acquired pneumonia (NV-HAP) and NV-HAP–associated sepsis and mortality outcomes. **Methods:** Havasu Regional Medical Center (HRMC) is a 171-bed acute-care community hospital in Lake Havasu City, Arizona. The hospital inpatient units measured in this quasi-experimental study were the medical surgical telemetry ortho unit (MTSO), the intermediate care unit (IMC), and the ICU.

There were 30,838 hospital patient days in 2021. NV-HAP were captured as patients coded as an NV-HAP and being discharged in 2021. Sepsis was captured as sepsis being documented with the source being identified as a NV-HAP with a discharge date in 2021. Mortality was captured by coding of an NV-HAP and mortality with a time of death documented in 2021. **Results:** From January 1, 2021, to June 4, 2021, during the baseline period before the oral-care bundle was implemented, HRMC had 12,415 patient days and experienced a NV-HAP rate of 1.2 per 1,000 patient days and a sepsis rate of 0.56 per 1,000 patient days with the source documented as NV-HAP, and mortality rate of 0.32 per 1,000 patient days with a code of NV-HAP. HRMC used June 5, 2021, as their implementation period of the bundle, which included a new oral-care device, multilevel education to staff on best practices for oral care, and daily audits to measure compliance with oral-care best practices. During the postimplementation period, HRMC had 18,413 patient days, a NV-HAP rate of 0.54 per 1,000 patient days, a sepsis rate of 0.33 per 1,000 patient days with source documented as NV-HAP, and a mortality rate of 0.16 per 1,000 patient days with a code for NV-HAP. **Conclusions:** From June 5, 2021, to December 31, 2021, after the implementation of the oral-care bundle, the NV-HAP rate decreased by 58%, the sepsis rate with source documented as NV-HAP decreased by 41%, and the morality rate documented as NV-HAP decreased by 50%. Hospital infection control programs should consider implementation of a robust oral-care bundle that includes best-practices education and auditing to monitor staff compliance as a potential strategy to reduce NV-HAP.

**Funding:** None

**Disclosures:** None

